# The Effect of Mother-Infant Skin to Skin Contact after Birth on Third Stage of Labor: A Systematic Review and Meta-Analysis

**Published:** 2019-04

**Authors:** Fatemeh Zahra KARIMI, Hamid HEIDARIAN MIRI, Maryam SALEHIAN, Talat KHADIVZADEH, Mohaddese BAKHSHI

**Affiliations:** 1. Nursing and Midwifery Care Research Center, Mashhad University of Medical Sciences, Mashhad, Iran; 2. Department of Biostatistics and Epidemiology, School of Public Health, Mashhad University of Medical Sciences, Mashhad, Iran; 3. Department of Midwifery, School of Nursing and Midwifery, Quchan Branch, Islamic Azad University, Quchan, Iran

**Keywords:** Skin to skin contact, Third stage of labor, Systematic review, Meta-analysis

## Abstract

**Background:**

One of the causes of postpartum hemorrhage is prolongation of third stage of labor. Mother-infant skin to skin contact (SSC) immediately after delivery is one of the non-pharmacological interventions to reduce this stage. Studies which assessed the effect of mother-infant SSC after delivery on duration of the third stage of labor reported controversial results on this issue. Therefore, this study investigated the effect of mother-infant SSC immediately after birth on the duration of third stage of labor

**Methods:**

In this systematic review and meta-analysis, the databases of PubMed, Scopus, Cochrane, SID, Magiran IranDoc and Google Scholar were searched from 2000 to 2018, using the keywords related to the objectives of this review to access randomized control trials published in Persian or English. The quality of papers was examined using Cochran's Risk of bias tool. Data was analyzed using Stata software. We used I2 index and Chi-square test to investigate heterogeneity and Egger’s and Begg’s tests to assess publications bias. Random effects model was used to combine the data.

**Results:**

Six studies were entered into the meta-analysis. The third stage of labor in SSC group was shorter than that of control group with a mean difference of −1.33 and 95% CI (−2.31 to −0.36) and this difference was statistically significant (*P*=0.007).

**Conclusion:**

Mother-infant SSC decreases the duration of third stage of labor. Therefore, the current study provides some evidences to use this non-pharmacological method in order to accelerate the third stage of labor and ultimately prevent postpartum hemorrhage.

## Introduction

Postpartum hemorrhage is one of the most common causes of maternal death worldwide. According to Centers for Disease Control and Prevention (CDC), hemorrhage was the direct cause of about 13% of 4693 pregnancy-related maternal deaths in the United States. In developing countries, the role of hemorrhage in maternal death is also higher, so that hemorrhage accounts for about half of all postpartum deaths in these countries (1, 2).

Uterine atony (uterus disability for sufficient contraction) and prolongation of third stage of labor could increase the risk of postpartum hemorrhage. The third stage of labor begins immediately after the birth of fetus and ends with the exit of placenta. One of the most important purposes in this phase is avoiding postpartum hemorrhage. Uterotonic factors like Oxytocin are the most important factors for reduction of postpartum hemorrhage; because they increase uterine contractions and accelerate the third stage of labor.

Today, immediate administration of synthetic oxytocin is the most important medical intervention to prevent postpartum hemorrhage. Maternal oxytocin strengthens the uterine contractions, which subsequently helps the uterus contraction and removing placenta from the uterine wall, and ultimately can prevent postpartum hemorrhage (1, 3, 4). While having high efficiency, synthetic drugs have adverse effects and this issue has raised the need for some other methods with less side-effect (5).

One of these methods, examined in the studies, is mother-infant Skin to Skin Contact (SSC) at the initial moments after delivery, in which the baby after birth is immediately placed in the middle of the mother's chest in the vertical position (6, 7). Mother-infant SSC is physiologically, psychologically, and clinically beneficial for mother and her baby. During SSC, contact, heat, and olfactory receptors which have strong vagus nerve stimulants can lead to the release of maternal oxytocin. Oxytocin is one of the most important uterotonic factors and plays an important role in uterus contraction, acceleration of the third stage of labor and controlling postpartum hemorrhage. Oxytocin also has anti-anxiety effects and can increase the feeling of comfort and confidence; it has a very important role in mammals on the onset of maternal behaviors. Moreover, SSC leads to the development of nutritional behaviors in the newborn; consequently, the baby takes the mother's breast and gains the ability to suck and begins to nourish (8–12).

Although there are reports on the benefits of mother-infant SSC, some studies reported conflicting results about the effect of mother-infant SSC on third stage of labor. The early mother-infant SSC after birth significantly decreased the time of placenta separation and the duration of third stage of labor (6, 13, 14). While there was no significant association between mother-infant SSC and the duration of third stage of labor (15). Therefore, due to inadequacy of evidence in this area and controversy of results of available studies, it is necessary to conduct a systematic and critical review of the available studies to provide a clear guide for policy makers and researchers.

The current study was conducted to evaluate the effect of mother-infant SSC immediately after birth on the duration of third stage of labor.

## Materials and Methods

In this systematic review and meta-analysis, all studies on the effect of mother-infant SSC on the third stage of labor were recovered using the keywords “Third stage of labor OR Placenta separation OR delivery OR placental delivery) AND (skin to skin contact OR skin to skin mother-infant contact OR SSC OR Kangaroo Mother Care Methods OR Kangaroo Mother Care OR KMC) AND (Randomized Clinical Trials)” and using boolean OR and AND operators in databases of SID, Magiran, Irandoc, Scopus, Pub-Med, ISI Web of Science, Cochrane, Google Scholar from 2000 to August 2018. The PRISMA flowchart was used to report the process of studies selection.

The inclusion criteria for meta-analysis were: 1- Clinical trials published in Persian or English examined the effect of mother-infant SSC on the duration of the third stage of labor, 2- The participants consisted of Mothers and term healthy infants (between 37 and 42 wk of pregnancy). 3- The intervention consisted of a SSC that defined as placing a naked newborn infant in the prone of mother's bare chest immediately after birth, 4- the comparison consisted of routine care, 5- the primary outcome was duration of the third stage of labor 6- time points of measurements was third stage of labor, this stage begins immediately after the birth of the fetus and ends with the exit of the placenta. There was no secondary outcome included. The articles with incomplete and non-relevant data were excluded from the meta-analysis.

To select the studies in the first stage, the title, abstract and keywords and their eligibility were evaluated. In the second stage, the full text of selected studies were independently reviewed by two authors for further assessment of eligibility and were discussed until reaching consensus.

**Fig. 1:**
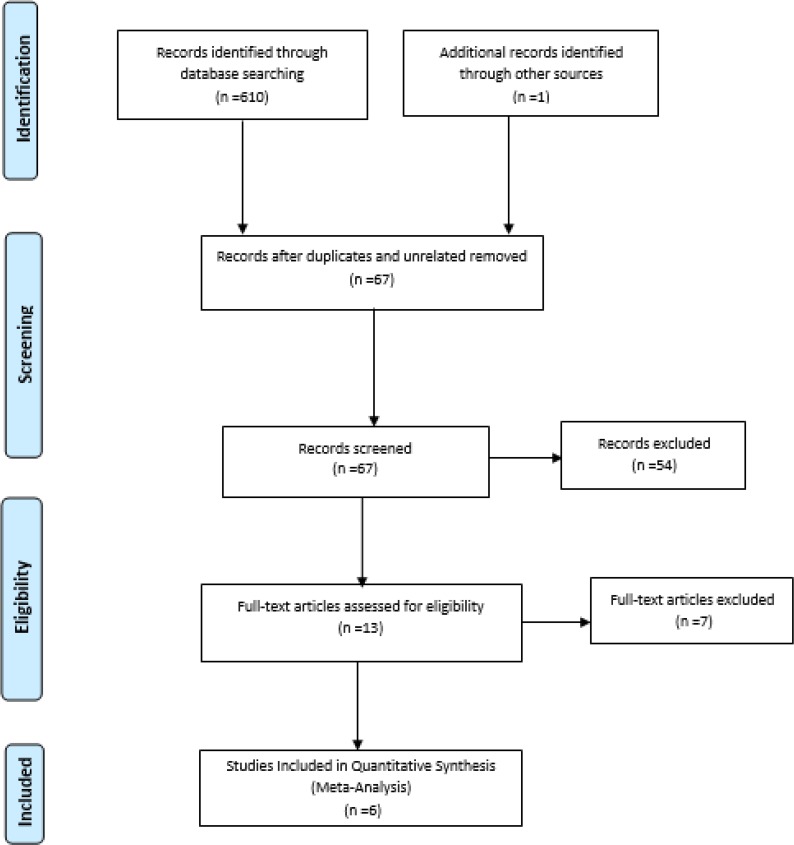
PRISMA Flowchart of the study selection process

Through searching in various databases, 611 articles were found. Then, 544 were removed because they were either repetitive or unrelated. Next, the remaining 67 studies were evaluated based on the title and abstract from which 54 papers were not qualified for full text assessment. Then, the full text of the 13 qualified papers were examined from which 7 articles were excluded due to incomplete and irrelevant results and 6 papers were entered the meta-analysis. Flowchart of studies review is shown in Fig. 1.

Risk of bias of each study was examined by two independent evaluators using the Cochrane bias checking tool and the extracted data were recorded in the relevant forms. In the absence of an agreement between the two evaluators, the issue was examined and discussed by the presence of an observer until reaching consensus. This tool examines risk of 6 types of biases including selection bias (random sequence generation, allocation concealment), Performance bias (examining the blinding of participants and personnel), detection bias (Blinding of outcome assessment assessors), attrition bias (Incomplete outcome data), the reporting bias (the selective reporting), and other sources of biases. Based on the degree of bias, studies with low, high and uncertain risks were considered and reported for each part (16, 17) (Table 1).

**Table 1:** Characteristics of 6 clinical trials included in study

***No.***	***Author*** ***Year*** ***Location of the study***	***Participants***	***Intervention***	***Comparison***	***Instrument***	***Outcome***
1.	Safari et al 2018 Iraq	Mother-infant pairs N=108	Mother-infant SSC N=56	Routine care N=52	Stopwatch	Duration of the third stage of labor in SSC group 6±1.7 min, vs 8.02±3.6 min for routine care group (*P*<0.001).
2.	Khadivzadeh et al 2018 Iran	Mother-infant pairs N=92	Mother-infant SSC N= 47	Routine care N= 45	Chronometer	There was no significant difference in the mean duration of the third stage of labor between the groups (*P*=0.3)
3.	Al-Morbaty et al 2017 Saudi Arabia	Mother-infant pairs N=28	Mother-infant SSC N=14	Routine care N=14	Stopwatch	The mean duration of placental delivery was (441.4±303.7 sec) and (550.4± 290.4 sec) in the experimental and control groups Respectively (*P*=0.042)
4.	Essa and Ismail 2015 Egypt	Mother-infant pairs N=100	Mother-infant SSC N=50	Routine care N=50	Timer	The mean duration of the third stage of labor in the study group was significantly shorter (2.8±0.857 min) than among those in the control group (11.22±3.334 min) (*P*<0.01).
5.	Marín Gabriel et al. 2014 Iran	Mother-infant pairs=350 N=90	Mother-infant SSC N=137	Routine care N=137	Timer	Mean time to expel the placenta was 408.7±244.8 sec in the SSC group vs 475.2±276.6 sec in the CG (*P*=0.05).
6.	Mejbel et al 2012 Iraq	Mother-infant pairs N=80	Mother-infant SSC N= 40	Routine care N= 40	Timer	There are significant differences between SSC and CG of placental separation time (mean 1.880±-.65758 vs 8.0750±2.76783; *P*=0.000).

To extract the data, two authors independently extracted the data from the full text of all the final papers included in the study process with a pre-prepared checklist. The checklist included the names of the authors, publication year, the type of study, the sample size, the scale used, the results, and the risk of bias (using the Cochrane check list).

Meta-analysis was performed using Stata sofware version 14.1 and with using mean, standard deviation and sample size of studies. To adjust for differences in scales, means were standardized based on Cohen method. The I2 index was used for examining the homogeneity among the studies and the Egger’s and Begg’s tests were applied to investigate the possibility of publication bias.

Due to heterogeneity in the studies, a random effects model was used to perform meta-analysis.

## Results

**Fig. 2:**
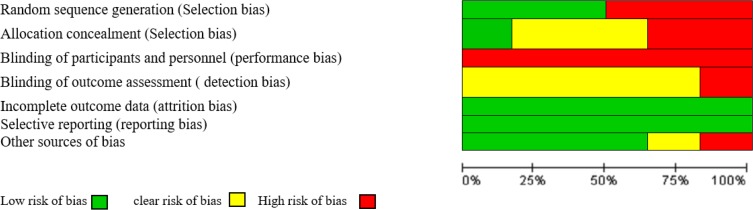
Risk of bias graph: Systematic review. Author’s judgments of risk of bias presented as percentages across all included studies

In the databases search, 611 articles were first obtained; in the selection process, 6 studies were entered the meta-analysis (Fig. 1). Of the six studies, one study was conducted in Iran, one in Saudi Arabia, two in Iraq, and one in Spain. Assessing the third stage of labor was done with stopwatch or timer. All studies were written in English. Data of each of the studies included in the quantitative analysis process are presented in Table 1. The results of investigating the risk of bias in studies using the Cochrane checklist are shown in Table 2 and Fig. 2.

**Table 2:** Risk of bias summary: Systematic review. Author’s judgments of risk of bias item for each included study (NA: Not applicable)

***Reference***	***Random sequence generation (Selection bias)***	***Allocation concealment (Selection bias)***	***Blinding of participants and personnel (performance bias)***	***Blinding of outcome assessment (detection bias)***	***Incomplete outcome data (attrition bias)***	***Selective reporting (reporting bias)***	***Other sources of bias***
Safari et al 2018	+	?	−	?	+	+	+
Khadivzadeh et al. 2016	+	+	−	?	+	+	+
Al-Morbaty et al 2017	+	?	−	?	+	+	+
Essa and Ismail 2015	−	−	−	?	+	+	**+**
Mejbel et al 2012	−	?	−	?	+	+	−
Gabriel et al. 2009	−	−	−	−	+	+	?

**Fig. 3:**
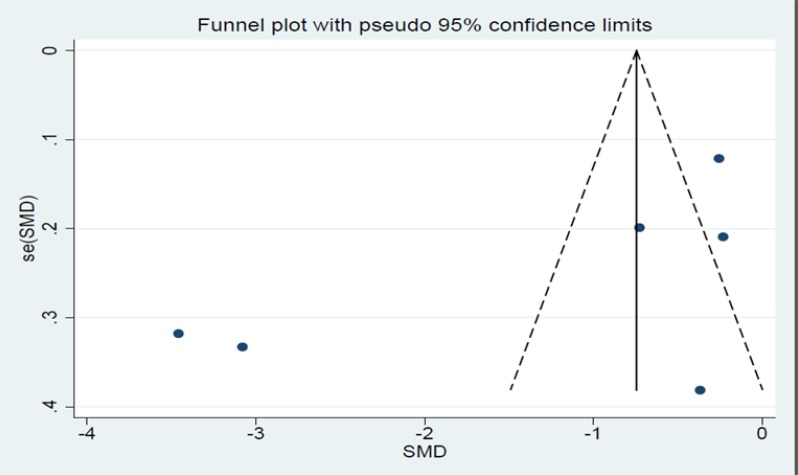
Funnel plot for publication bias evaluation

Regarding publication bias, *P-*value of Egger’s and Begg’s was 0.13 and 0.45 respectively which indicates the hypothesis that there is no publication bias and the corresponding funnel plot is symmetry cannot be rejected (Fig. 3).

**Fig. 4:**
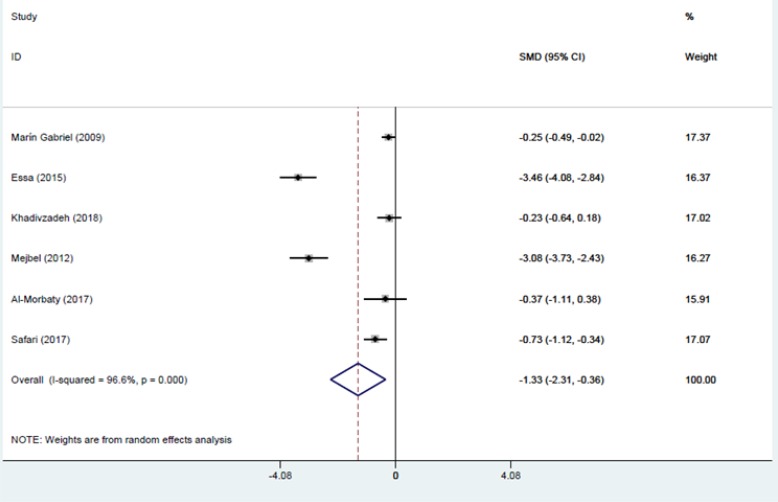
Standardized mean difference (SMD) as −1.33 with 95% CI from −2.31 to −0.36 (*P* value=0.007), indicates that Mother-Infant Skin to Skin Contact after birth decreases the duration of the third stage of labor. The horizontal lines denote the 95% CI, the Square (▪) shows the point estimate (the size of the square corresponds to its weight); the diamond shows (◊) the combined overall effects of treatments

Considering heterogeneity, I^2^ was 96.6% (*P*=0.00) which shows that there is some real differences among findings of the included studies and as a result random effects model was used to account for this differences (Fig. 4).

Figure 4 shows the findings of all included studies as well as the summary effect both graphically and numerically. The midpoint of each line and the length of the line respectively show the mean difference and its 95% confidence interval for each study. The size of squares represent the weight which that study had on the overall summary effect. The middle of the diamond sign shows the summary effect and the horizontal diameter of it represent 95% confidence intervals of the summary effect. Mother-infant SSC can significantly reduce the duration of the third stage of labor by mean difference (MD) of −1.33 with a 95% confidence interval from −0.36 to −2.31 (*P*=0.007).

## Discussion

In the current systematic and meta-analysis review, six controlled randomized clinical trials examined the effect of mother-infant SSC on the duration of third stage of labor were reviewed. The results of the present meta-analysis showed that mother-infant SSC significantly reduces the mean duration of third stage of labor compared with the conventional care.

Many studies have been conducted on the effect of mother-infant SSC on the duration of third stage of labor, but so far no meta-analysis has been done in this area. Among the studies examined the effect of mother-infant SSC on the duration of third stage of labor, the studies showed that mother-infant SSC reduces the mean of third stage of labor in the intervention group compared to the conventional care group, and significant difference was found between two groups (6, 13, 14, 18). While in other studies, no statistically significant difference was observed between two groups of receiving SSC and conventional care in terms of mean duration of third stage of labor (15, 19).

One of the most important objectives of meta-analysis studies is providing an accurate and valid result due to the increased sample size because of the combination of various studies, and thus reducing the confidence intervals and solving the problems arising from the controversial results of past studies. In addition, these types of studies can provide the best evidence for judging about the impact of interventions in medicine and its use in the clinic (20).

About the mechanism of the effect of mother-infant SSC on the duration of third stage of labor, during skin contact, when the baby touch the mother's body, oxytocin hormone secretes from the posterior pituitary and leads to increased level of maternal oxytocin.

Moreover, during mother-infant SSC, the verbal and contact interaction increases between mother and her infant which could lead to an increase response in the mother's body stimulations that finally results in the development and progression of nutritional behaviors through smell in the baby, so the baby takes the mother's breast and gains the ability to suck.

When the baby stimulates the mother’s breast and begins to suck, the level of endogenous oxytocin increases, because mother's breast suction stimulates the neurohypophysis to release oxytocin. During suction, stimulation of the nerve terminal of the areola lead to oxytocin release from the posterior pituitary. Oxytocin hormone is very prominent during labor because of its role in the uterine contraction. In addition, when the baby is placed in skin contact with mother, the movements of the baby's feet on the mother’s abdomen acts like uterine massage which can stimulates the uterine contractions and accelerates separation and exit of placenta and ultimately reduces postpartum hemorrhage (4, 6, 19, 21–26).

Postpartum hemorrhage is one of the main causes of maternal mortality and morbidity for which uterine atony and prolongation of third stage of labor are two of the commonest causes of postpartum hemorrhage. Therefore, prevention of atony and reduction of duration of third stage of labor are the best measures to prevent postpartum hemorrhage; the first step in prevention and treatment is administration of uterine contraceptive drugs (1–3). Synthetic oxytocin is the most commonly used treatment which while having high efficiency have adverse effects and this has raised the need for replacement with less harmful methods (5). The present study and other studies have shown that mother-infant SSC immediately after delivery can be used as a relatively easy, inexpensive, and noninvasive method to increase the production of endogenous oxytocin. In addition, the stimulation to the mother's body caused by the baby during skin contact can increase the uterine contractions.

The results of this study can be used by health care providers and be integrated into maternal and neonatal care programs. As mother-infant SSC is still not performed optimally in Iran and the mother and her baby are separated after birth in many cases in order to perform the hospital usual practices. It is recommended the service providers be trained about the importance of the mother-infant SSC after childbirth and even be obliged to implement it.

One of the strengths of the present study was that it is the first systematic review and meta-analysis which investigated the effect of mother-infant SSC on the duration of third stage of labor.

## Conclusion

Mother-infant SSC decreases the duration of third stage of labor. Therefore, it can be used by policy makers and service providers in evidence-based decision-making in the field of maternal and neonatal health. Therefore, since postpartum hemorrhage is one of the most common causes of maternal death in the world, we can accelerate the third stage of labor and prevent postpartum hemorrhage through mother-infant SSC which is a simple and inexpensive method without any side effects and by delaying the conventional care measures until the end of skin contact. It is suggested that contact between the mother and the baby be adopted as a care method by maternal and child health care providers, such as midwives, doctors, and students responsible in childbirth.

## Ethical considerations

Ethical issues (Including plagiarism, informed consent, misconduct, data fabrication and/or falsification, double publication and/or submission, redundancy, etc.) have been completely observed by the authors.
